# Genetic Diversity and Forensic Parameters of 27 Y-STRs in Two Mestizo Populations from Western Mexico

**DOI:** 10.3390/genes16030352

**Published:** 2025-03-19

**Authors:** Astrid Desireé Sánchez-Méndez, Silvia Elena Narvaez-Rivera, Héctor Rangel-Villalobos, Jorge Hernández-Bello, Andrés López-Quintero, José Miguel Moreno-Ortíz, Benito Ramos-González, José Alonso Aguilar-Velázquez

**Affiliations:** 1 Doctorado en Genética Humana, Centro Universitario de Ciencias de la Salud, Guadalajara 44340, Jalisco, Mexico; 2 Laboratorio de Ciencias Morfológico Forenses y Medicina Molecular, Departamento de Morfología, Centro Universitario de Ciencias de la Salud, Guadalajara 44340, Jalisco, Mexico; 3 Instituto de Criminalística y Servicios Periciales, Fiscalía General de Justicia del Estado de Nuevo León (FGJNL), Monterrey 64649, Nuevo León, Mexico; 4 Instituto de Investigación en Genética Molecular, Centro Universitario de la Ciénega, Universidad de Guadalajara, Ocotlán 47810, Jalisco, Mexico; 5 Instituto de Investigación en Ciencias Biomédicas, Centro Universitario de Ciencias de la Salud, Universidad de Guadalajara, Guadalajara 44340, Jalisco, México; 6 Instituto de Nutrigenética y Nutrigenómica Traslacional, Centro Universitario de Ciencias de la Salud, Universidad de Guadalajara, Guadalajara 44340, Jalisco, Mexico; 7 Instituto de Genética Humana “Dr. Enrique Corona Rivera”, Departamento de Biología Molecular y Genómica, Centro Universitario de Ciencias de la Salud, Universidad de Guadalajara, Guadalajara 44340, Jalisco, Mexico

**Keywords:** Mexican Mestizos, genetic diversity, forensic parameters, Y-STRs, Yfiler™ Plus

## Abstract

Background: Analyzing Y-chromosome short tandem repeats (Y-STRs) is essential in forensic genetics and population studies. The Yfiler™ Plus kit, which includes 27 Y-STR markers, enhances the discrimination power for forensic and kinship applications. However, this genetic system has not been analyzed in Mexican populations, which limits its application and representativeness in international databases. Objectives: We wished to examine the genetic diversity and forensic parameters of the 27 Y-STRs included in the YFiler™ Plus kit in two populations from Western Mexico (Jalisco and Michoacán). Methods: Male DNA samples were amplified using the Yfiler™ Plus kit, followed by a fragment analysis via capillary electrophoresis (CE). The haplotype frequencies and forensic parameters were calculated. The haplogroups of all samples were predicted, and the distribution and percentages of ancestries were determined. The Rst genetic distances, including reference populations, were calculated and graphically represented in a multidimensional scaling (MDS) plot. Results: A total of 224 haplotypes were identified in all of the samples, of which 98.66% corresponded to unique haplotypes. Bi- and tri-allelic patterns were observed in both populations. The observed discriminatory capacity was 98.4% for Jalisco and 98.9% for Michoacán, while the haplotype diversity values were 0.9998 and 0.9997, respectively. The most frequent haplogroup was R1b, followed by Q, representing the European and Native American ancestries, in both populations. Conclusions: This study is the first to report the haplotype diversity and forensic parameters of the 27 Y-STRs included in the Yfiler™ Plus kit in Mexican populations. These findings confirm the forensic utility of these markers for human identification, biological relationship testing, and criminal investigations, reinforcing their applicability in forensic casework.

## 1. Introduction

Over 500 years of intermixing between Native Americans, European conquerors (primarily Spaniards), and Africans led to the formation of most of the contemporary Mexican population (~93%), commonly known as Mestizo [1,2]. The remainder of the Mexican population consists of Native American groups, who arrived at least 23,000 years ago and are descendants of the first settlers of the Americas [3].

Early research on Mexican Mestizos proposed a trihybrid model to account for their biological diversity, with distinct ancestries more prominent in different regions: European in the north, Amerindian in the center and southeast, and African along the coasts [4]. However, later studies using short tandem repeat (STR) loci have revealed a contrasting genetic pattern across Mexico, where Native American ancestry increases gradually from north to south. In contrast, European ancestry intensifies from south to north [1,5]. Interestingly, this pattern has been suggested to reflect the pre-Hispanic Mesoamerican demography [1] or to recapitulate the Native American substructure [6].

STRs linked to the Y-chromosome (Y-STR) are genetic markers inherited exclusively through the paternal lineage. They are present in a haploid state and, except for those located in the pseudo-autosomal regions, do not undergo recombination. These properties make Y-STRs a useful tool in sexual assault investigations, paternity and genealogical testing, disaster victim identification, and evolutionary studies [7,8,9]. Despite their forensic relevance, Y-STRs analyses remain limited in Mexican Mestizo populations with only a few studies reported [10,11,12]. Most studies have focused on 9, 12, and 17 Y-STRs, and only two studies have been based on 23 markers (PowerPlex Y23) [13,14]. No studies have been conducted using the Yfiler™ Plus PCR Amplification Kit (Thermo Fisher Scientific; Waltham, MA, USA), which allows for the analysis of 27 Y-STR markers [15]. This kit includes the 17 loci found in the Yfiler^®^ kit (DYS19, DYS385a/b, DYS389I/II, DYS390, DYS391, DYS392, DYS393, DYS437, DYS438, DYS439, DYS448, DYS456, DYS458, DYS635, and Y-GATA-H4), along with 3 highly polymorphic Y-STR loci (DYS460, DYS481, and DYS533) and 7 rapidly mutating (RM) Y-STR loci (DYF387S1a/b, DYS449, DYS518, DYS570, DYS576, and DYS627). Due to their high mutation rate, RM Y-STRs are particularly useful in complex cases where distinguishing individuals from the same paternal lineage is necessary.

Analyzing genetic systems such as YFiler™ Plus is crucial in Mexican populations from states such as Jalisco and Michoacán, which hold significant economic and cultural relevance but also exhibit some of the country’s highest crime and delinquency rates. The lack of extensive Y-STR databases for Mexican populations poses challenges in forensic casework, particularly in regions with high levels of criminal activity, where genetic markers play a crucial role in individual identification. Therefore, this study aimed to analyze the genetic diversity and forensic parameters of the 27 Y-STRs included in the Yfiler™ Plus kit in Jalisco and Michoacán population samples.

## 2. Materials and Methods

### 2.1. Sample Collection and DNA Extraction and Quantification

Overall, 224 blood samples were collected on Whatman FTA cards (Cytiva, Marlborough, MA, USA) from individuals from the states of Jalisco (*n* = 129) and Michoacán (*n* = 95) in western Mexico. In compliance with the ethical guidelines of the Helsinki Declaration, all of the participants voluntarily consented to participate in this study and provided written informed consent. The Ethics, Research, and Biosafety Committees of the Centro Universitario de Ciencias de la Salud approved the project at the Universidad de Guadalajara (approval code: CI-04924; approval date: 15 August 2024). The anonymity of all individuals was strictly maintained throughout this study. DNA was extracted from each sample’s FTA paper punch (Whatman^®^) using the PrepFiler™ Express BTA kit (Thermo Fisher Scientific, Waltham, MA, USA) following the supplier’s instructions. DNA was quantified using the Quantifiler^®^ Trio DNA quantification kit (Thermo Fisher Scientific, Waltham, MA, USA) in a 7500 Applied Biosystems real-time PCR system (Applied Biosystems, Thermo Fisher Scientific, Waltham, MA, USA).

### 2.2. Amplification and Fragment Analyses

Amplification of the 27 Y-STR loci was performed by employing the Yfiler™ Plus PCR Amplification Kit (Thermo Fisher Scientific, Waltham, MA, USA) on a PCR ProFlex™ Thermal Cycler (Thermo Fisher Scientific, Waltham, MA, USA) according to the supplier’s instructions. A fragment analysis was performed using capillary electrophoresis on a SeqStudio genetic analyzer (Thermo Fisher Scientific, Waltham, MA, USA). Further, the results were analyzed using the GeneMapper ID-X v.1.6 software, according to the reference allelic ladder.

As specified in the HID system, positive and negative controls were used. Two analysts verified the Y-STR haplotype data independently. The laboratory where the samples were processed annually participates in a quality control proficiency test organized by the Latin American Society for Forensic Genetics (http://slagf.org.ar/, accessed on 1 January 2024).

### 2.3. Statistical Analysis

The haplotype frequency was determined using a direct counting approach. The haplotype diversity (HD) was calculated using the formula *HD* = *n* * (1 − ∑ *pi*^2^)/(*n* − 1), where *n* represents the population size and *pi* denotes the frequency of the *i*^-th^ haplotype. The haplotype match probability (HMP) was determined by summing the squares of the observed haplotype frequencies. The discrimination capacity (DC) was computed as the ratio of the number of distinct haplotypes to the total number of haplotypes. The haplotype match probability (HMP) was calculated as the sum of square observed haplotype frequencies. The discrimination capacity (DC) was calculated as the ratio between the total distinct haplotypes and the number of haplotypes. The genetic distances (Rst) and *p*-values were calculated and graphically represented on an MDS plot employing the tools available in YHRD (https://yhrd.org/, accessed on 1 January 2025), including the following populations as references: Puebla (Mexico) [unpublished data], Costa Rica [16], Ecuador [17], Peru [18], Spain [19], and African Americans, European Americans, and Native Americans from the U.S. [20]. Haplogroup prediction was performed using the NevGen Y-DNA Haplogroup Predictor [21], which estimates the probability of the haplogroup affiliation for a given Y-STR haplotype. The haplogroup assigned corresponds to that with the highest probability. This predictor was selected due to its previously demonstrated accuracy in haplogroup classification [22,23]. Studies have shown that when at least 20 Y-STR markers are analyzed, the probability of correctly predicting the Y-haplogroup exceeds 99% in nearly all cases [24]. For assignment of the haplogroup distribution, we adhered to the guidelines of the International Society of Genetic Genealogy (ISOGG: https://isogg.org/tree/2018/index18.html, accessed on 1 January 2025) and the minimal reference phylogeny for the human Y chromosome [25].

## 3. Results

### 3.1. Haplotype Database Description

Since Y-STRs do not undergo recombination, haplotype data more accurately represent population diversity. A complete Y-STR haplotype dataset for the Jalisco and Michoacán populations is available in Table S1 and has been included in release 70 of YHRD (submission numbers: Jalisco, YA006057; Michoacán, YA006058). The haplotype frequencies are provided in Table S2. A total of 129 haplotypes were identified from Jalisco and 95 from Michoacán. Interestingly, bi- and tri-allelic patterns were observed in both populations. In Jalisco, one haplotype exhibited bi-allelic patterns at the DYS448 (alleles 19/20) and DYS570 (alleles 19/26) markers. In contrast, in the Michoacán population, two tri-allelic patterns were identified at the DYS456 (alleles 15/16/17) and DYS19 (alleles 14/15/16) markers, while a bi-allelic pattern was detected at DYS438 (alleles 10/12).

### 3.2. Forensic Parameters

No dropout or null alleles were observed. The haplotypes generated using the Yfiler™ Plus kit exhibited a high number of unique haplotypes per population, with 127 in Jalisco and 94 in Michoacán; these significant numbers of different haplotypes generate discriminatory capacity (DC) values of 98.6 and 98.9%, respectively. The haplotype diversity (HD) was 0.9998 for Jalisco and 0.9997 for Michoacán. In contrast, the haplotype match probability (HMP) values were 0.00793222 for Jalisco and 0.010747922 for Michoacán.

### 3.3. Genetic Distances

**Figure 1:**
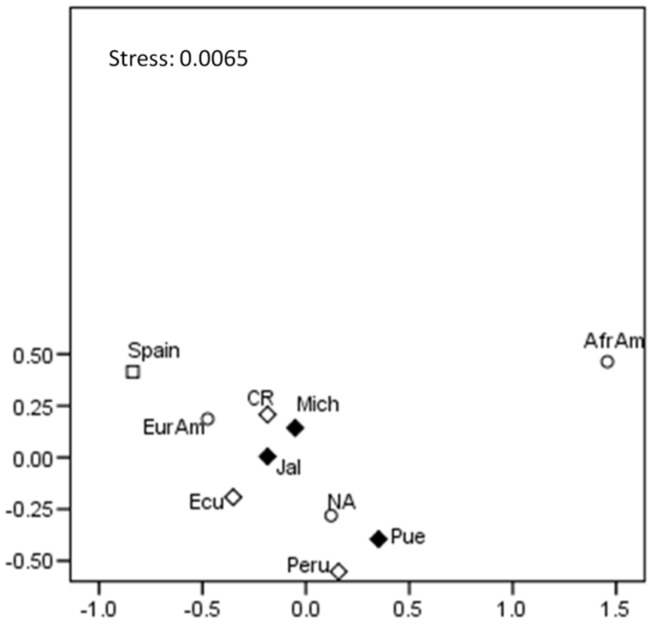
MDS plot based on Rst distances between Mestizo and world populations based on 27 Y-STRs. Rhombi: Mestizo populations from the Americas; black rhombi: Mexican Mestizo populations; circles: major U.S. groups; squares: Spaniards. Jal: Jalisco; Mich: Michoacán; Pue: Puebla; CR: Costa Rica; Ecu: Ecuador; NA: Native American; AfrAm: African American; EurAm: European American.

The Rst genetic distances and *p*-values are available in Table S3. Notably, Jalisco and Michoacán exhibited close but non-significant genetic distances (Table S3). The Mexican populations exhibited relatively close genetic distances from other Mestizo populations in the Americas (Peru, Costa Rica, and Ecuador), as well as from Spanish populations and U.S. groups of European, African, and Native American descent. However, the differences among the Costa Rican, Ecuadorian, and European descendants were not significant (Table S3). As shown in the MDS plot (Figure 1), the Mexican populations of Jalisco and Michoacán, as well as the Costa Rican population, are positioned closer to the U.S. group of European descent. In contrast, the populations of Puebla (south–central Mexico), Peru, and Ecuador are closer to the Native American sample from the U.S., highlighting the predominant ancestries in Mestizo populations across the Americas.

### 3.4. Haplogroup Distribution

**Figure 2:**
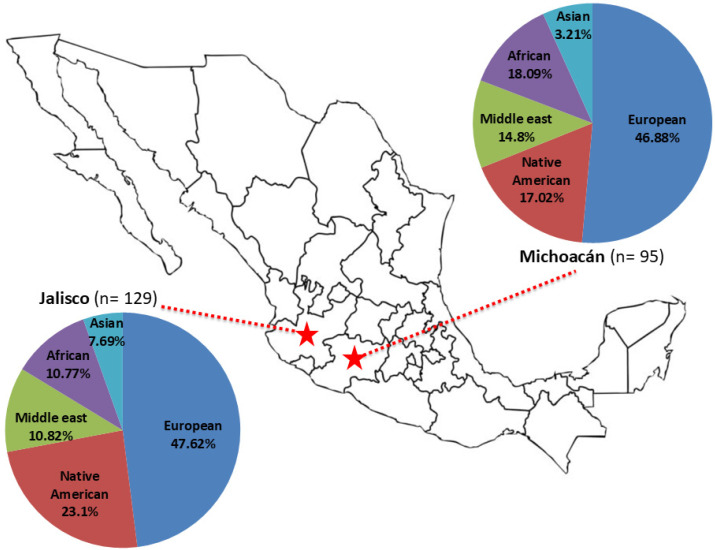
Geographic localization of Jalisco and Michoacán states in Mexico and haplogroup ancestry distribution reported in this study.

Overall, twenty-one haplogroups were identified: R1b, R1a, I2a1a, I1, I2a2a, I2a1, Q, J2a1, T, J1a, J1a2a1a2, E1b1b, E1b1a, E1a, G2a2b2a1c, G2a2b2a1b, O2a1, N1a1, G2a2, G2a2a, and G2a1. The most frequent haplogroup was R1b (40.9% in Jalisco and 37.3% in Michoacán), followed by Q and E1b1b, in both populations (Table 1). Regarding ancestry, the highest proportion was European in both populations (47.62% in Jalisco and 46.88% in Michoacán), followed by Native American (23.1% in Jalisco and 17.02% in Michoacán), Middle Eastern (11.63% in Jalisco and 12% in Michoacán), African (10.77% in Jalisco and 18.09% in Michoacán), and Asian (7.69% in Jalisco and 3.21% in Michoacán) (Table 1, Figure 2).

## 4. Discussion

The genetic diversity and forensic statistical parameters characterized in this study highlight the significant utility of the Yfiler™ Plus kit in the Mexican populations of Jalisco and Michoacán. The results exceed the previously reported haplotype diversity and discrimination capacity values of the markers included in the PowerPlex Y, YFiler, and PowerPlex Y23 kits [10,11,12,13,14]. Notably, only three repeated haplotypes were identified across the entire database analyzed in this study, in contrast to studies on the Monterrey population (in the northeast region of Mexico) based on 23 Y-STRs (PowerPlex Y23), where many repeated haplotypes were found among unrelated individuals (375/400; DC = 93.75) [13]. This finding underscores the robustness of the Yfiler™ Plus genetic system.

Additionally, the bi- and tri-allelic patterns observed in both populations occur at very low frequencies in the STRBase database (https://strbase-archive.nist.gov/, accessed on 1 January 2025). Specifically, the bi-allelic pattern 19/20 in DYS448 has a reported frequency of 1%, while the 19/26 alleles in DYS570 occur at a frequency of 1–2%, and the 10/12 alleles in DYS438 are found at 0.01–0.03%. In contrast, the tri-allelic patterns in DYS456 (15/16/17) and DYS19 (14/15/16) have frequencies of 0.01–0.03% and 0.2%, respectively. These rare allele patterns have been associated with chimerism or structural mutations such as chromosomal segment deletions, duplications, or gene conversion [26]. However, these rare patterns could be valuable for human identification purposes. Moreover, in the future, marker patterns with copy number variations may become increasingly relevant and potentially critical in resolving forensic cases [27].

The genetic distance analyses indicate low differentiation between the Mexican populations studied and some admixed Central American populations. However, the lack of statistically significant results does not fully support this differentiation (Table S3). Conversely, the differentiation patterns observed in the multidimensional scaling (MDS) analysis (Figure 1) are consistent with previous studies on Mexican populations using autosomal STRs and Y-STRs, as well as genome-wide SNPs [1,5,6,28]. These studies have reported that populations from northern and western Mexico exhibit more European ancestry, whereas populations from central and southern Mexico display a more substantial Indigenous ancestral component.

Interestingly, this study inferred a greater number of haplogroups (21 vs. 17) compared to that in a previous study on Mexican populations [28], despite the latter using a more extensive database but a smaller number of markers (17 vs. 25 Y-STRs). This finding may support the idea that increasing the number of markers can enhance the accuracy of haplogroup predictions based on Y-STRs [22]. This is the first time the Michoacán population has been analyzed using Y-STRs, and it shows a slightly lower level of European ancestry (<1%) than that in Jalisco but higher levels of African and Middle Eastern ancestry. In comparison, Jalisco has a relatively higher level of Native American ancestry than that in Michoacán. This pattern is consistent with previous findings on Mexican populations [28,29].

Previous studies using Yfiler™ Plus across various global populations have reported a comparably high haplotype diversity and discriminatory capacity [18,20,30,31,32], similar to those observed in the populations of Jalisco and Michoacán. However, differences in the haplogroup distribution and allele frequencies highlight the unique genetic composition of each population, emphasizing the importance of region-specific forensic databases [7,8,9]. Future analyses with larger datasets could provide a deeper understanding of the genetic structure and forensic applicability of Y-STR markers across the different populations of Mexico.

Previous studies on Mexican populations using the 24 Y-STRs included in the ForenSeq DNA Signature Prep Kit have shown promising results, particularly in increasing the resolution of the forensic markers and enabling the detection of additional genetic variation based on the repetition sequence of the markers (repeat-sequence-based alleles) [27,33]. The ability of MPS to analyze sequence-based polymorphisms and provide deeper insights into haplotype diversity makes it a valuable tool for future forensic applications. However, Yfiler™ Plus remains a valuable alternative for identification due to its simpler and more accessible technique for most forensic laboratories (PCR-CE), and the RM Y-STRs included in this genetic system are especially useful in solving cases where it is necessary to differentiate males from the same paternal lineage.

Despite the broad utility of the Yfiler™ Plus kits, there have been no reports of their application in Mexican populations before this study, and their use in populations throughout the Americas has been minimal [18]. Including a greater variety of representative samples from diverse states or regions of Mexico would enhance the reliability of the forensic statistical parameters and improve the representation of admixed populations in international Y-STR databases. This is crucial to strengthening forensic applications, such as human identification and kinship analyses.

## 5. Conclusions

To the best of our knowledge, this study is the first to report the use of the 27 Y-STRs included in the Yfiler™ Plus kit in Mexican populations. Its results confirm the suitability of these 27 Y-STR markers for forensic applications in the populations of Jalisco and Michoacán, demonstrating their effectiveness in casework resolution. Additionally, the haplotypes reported in this study enhance the representation of Mexican populations in the YHRD international forensic database.

## Figures and Tables

**Table 1:** Ancestry and haplogroup distribution in Jalisco (*n* = 219) and Michoacán (*n* = 95).

	Jalisco		Michoacán	
Haplogroup	Frequency (%)	Ancestry	Frequency (%)	Ancestry
R1b	40.90	European (47.62%)	37.30	European (46.88%)
R1a	2.10		4.24	
I2a1a	1.55		1.07	
I1	1.55		3.20	
I2a2a	0.76		1.07	
I2a1	0.76		-	
Q	23.10	Native American (23.1%)	17.02	Native American (17.02%)
J2a1	5.43	Middle Eastern (10.82%)	7.40	Middle Eastern (14.8%)
T	3.10		-	
J1a	0.76		-	
J1a2a1a2	1.53		7.40	
E1b1b	8.46	African (10.77%)	17.02	African (18.09%)
E1b1a	1.55		1.07	
E1a	0.76		-	
G2a2b2a1c	3.10	Asian (7.69%)	-	Asian (3.21%)
G2a2b2a1b	0.76		-	
O2a1	1.55		-	
N1a1	0.76		1.07	
G2a2	0.76		-	
G2a2a	0.76		1.07	
G2a1	-		1.07	

## Data Availability

All of the data generated in this paper can be found in the digital version of the manuscript.

## Supplementary Materials

The supporting information can be downloaded at https://www.mdpi.com/article/10.3390/genes16030352/s1 as follows: Table S1. Haplotype database of Jalisco and Michoacan populations from Mexico. Table S2. Haplotype frequency in Jalisco and Michoacan populations from Mexico. Table S3. Genetic distances between Mexican and world populations.
