# Supplementary material for: Genetic Diversity and Forensic Parameters of 27 Y-STRs in Two Mestizo Populations from Western Mexico
Source: Genes (Basel). 2025 Mar 19;16(3):352. doi: 10.3390/genes16030352 (PMC11942575; doi:10.3390/genes16030352)
Supplement: Supplementary file 1 [file genes-16-00352-s001.zip › Table S3_Genetic distances.pdf]

Table S3. Genetic distances (Rst-value: above diagonal) and pairwise comparison (Fst *p*-values: below diagonal) between Mestizo populations from Mexico, Ecuador, Costa Rica and Peru, as well as Spaniards and USA populations (individuals with European and Native American ancestries) with the Y-STRs included in the YFiler Kit.

	Jal	Mich	CR	Ecu	Pue	Peru	Spain	AfrAm	EurAm	NA
**Jal**	-	0.6441	0.5042	0.0577	0.0000	0.0000	0.0000	0.0000	0.007	0.0000
**Mich**	-0.0025	-	0.1582	0.0089	0.0000	0.0000	0.0000	0.0000	0.0029	0.0000
**CR**	-0.0008	0.0035	-	0.0029	0.0000	0.0000	0.0000	0.0000	0.005	0.0000
**Ecu**	0.0078	0.0185	0.0178	-	0.0000	0.0000	0.0000	0.0000	0.0008	0.0000
**Pue**	0.0396	0.0485	0.0562	0.0445	-	0.0006	0.0000	0.0000	0.0000	0.0000
**Peru**	0.0395	0.0462	0.0568	0.0356	0.0085	-	0.0000	0.0000	0.0000	0.0000
**Spain**	0.0453	0.0555	0.0419	0.0466	0.1547	0.1462	-	0.0000	0.0002	0.0000
**AfrAm**	0.1638	0.154	0.1566	0.1962	0.1421	0.1495	0.2651	-	0.0000	0.0000
**EurAm**	0.0087	0.0155	0.0061	0.0193	0.0857	0.0849	0.0172	0.2044	-	0.0000
**NA**	0.0278	0.0369	0.0381	0.0321	0.012	0.0217	0.1129	0.1538	0.0553	-
